# The Tribe Scymnini (Coccinellidae: Coleoptera) From Sindh Province, Pakistan

**DOI:** 10.1093/jisesa/iev105

**Published:** 2015-10-10

**Authors:** Muhammad Ali, Rukhsana Perveen, Arif-Un-Nisa Naqvi, Khalil Ahmed, Ghulam Raza, Ishtiaq Hussain

**Affiliations:** ^1^Department of Biological Sciences, Karakoram International University, Gilgit, Gilgit-Baltistan, Pakistan; ^3^Department of Zoology, University of Karachi, Karachi, Pakistan; ^4^Department of Environmental Science, Karakoram International University, Gilgit, Gilgit-Baltistan, Pakistan

**Keywords:** Coccinellids, Scymnini, revision, IPM, Pakistan

## Abstract

Coccinellids are important natural enemies of aphids, scale insects, mealybugs, whiteflies, jassids and mites. They are being augmented or conserved for population reduction of different agricultural crop pests in the concept of Integrated Pest Management throughout the world. The genera and species in the tribe Scymnini known from Pakistan are revised and redescribed. Two genera including two subgenera and six species among which three species are newly reported, is therefore, a new addition to Coccinellid fauna of Pakistan. Keys to all taxa, descriptions of the higher taxa, species diagnoses, synonymies, and distribution records are included.

Members of the Scymnini are predaceous, feeding on homopterous insects (mostly aphids, adelgids, and scale insects); therefore, they are of interest to biological control. The tribe Scymnini composed of several genera and subgenera and is found throughout the world. The classification has always lagged behind that of other groups of Coccinellidae because the species are small, and accurate species identification usually depends on examination of male (Pang and Gordon 1986).

The coccinelids, *Nephus reguralis* Sicard, *Pseudoscymnus murreensis* Ahmad, *Pseudoscymnus* *simmondsi* Ahmad, *Scymnus* (*Pullus*) *coccivora* Ayyar, *S*. (*Neopullus*) *fuscatus* Boh and *S*. (*Scymnus*) *nubilus,* were recorded from Pakistan and Indian subregions (Poorani 2002).

Coccinellids housed in different institutions of Pakistan were studied and reported 162 species from different parts of the country except parts of the Baluchistan Province. Among these Coccinellids, 10 species were belonged to the tribe Scymnini (Hashmi and Tashfeen 1992). Thirteen species of predators were recorded associated with the mealy bug in agricultural fields at Multan, Tando Jam, and Winder (Baluchistan). These included Coccinellids were *Brumoides suturalis* (F.), *Scymnus coccivora* (Ayyar), *Scymnus* sp., *Nephus* sp., *Cheilomenes sexmaculatus* (F.), *Coccinella septempunctata* (L), *Hyperaspis* sp., *Adonia* sp., and *Exochomus* sp. (Mahmood et al. 2010). The following Coccinellids *N.* *reguralis* Sicard, *P. murreensis* Ahmad, *P*. *simmondsi* Ahmad, *Pullus coccivora* Ayyar, *Pseudoscymnus* *guimeti* Mulsant, *Pseudoscymnus* *pyrochaltus* Mulsant, *Scymnus guimeti* Mulsant, *S*. *fuscatus* Boh, *S*. *nubilus* were reported on members of the family Diaspidae, Pseudococcidae, Coccidae, Aphididae, Tetranychidae, and Adelgidae from Rawalpindi, Peshawar, Swat, Murree, Northern Areas, and Kashmir (Irshad 2001a,b, 2003; Irshad and Khan 2005; Rafi et al. 2005).

The male genitalia of the minute-sized species of the genus *Scymnus* Mulsant were treated for the first time in the history of the family Coccinellidae (Wilson 1927).

From Pakistan, very little taxonomic works have been noted. For the first time, two new species, *P.* *murreensis* Ahmad and *P*. *simmondsi* Ahmad, have been described from Northern parts of Pakistan (Ahmad 1966, 1968). Detailed morphological studies of different body parts of two species of the genus *Coccinella* L. have been done from Faisalabad region of the Punjab Province (Inayatullah and Siddiqui 1978, 1979; Inayatullah 1980). The taxonomy of 29 species of the family Coccinellidae including seven species of the tribe Scymnini were the latest and comprehensive works from the Sindh Province (Ali 2013). This study is also part this research carried out the Department of Zoology, University of Karachi, which deals with the redescription of the tribe Scymnini from Pakistan. This study present two genera, three subgenera, and total seven species with keys to the genera, subgenera, and species.

## Materials and Methods

Coccinellids were collected from different localities of the Sindh Province. The specimens were mounted after boiled in 10% solution of KOH for 10–15 min. Various body parts were separated and mounted in Canada balsam after a brief dip in xylol. Different structures including genitalia were studied under a Kruss Binocullar. Measurements and drawings of the body and other structures were made by using a micromillimeter scale and an ocular grid. The terminologies for various taxonomic structures including genitalia and procedures used by the following scientists were generally followed (Inayatullah and Siddiqui 1978, Gordon 1985). The taxonomic structures especially male and female genitalia after illustration were preserved in microvial with glycerine and pinned with specimen. All diagrams are to the given scales, and all measurements are in millimeters.

All specimens were identified by the author following the checklists, descriptions, and keys given by Chapin and Ahmad (1966), Pang and Gordon (1986), Poorani (2002), Rafi et al. (2005), and with the help of the following website: NBAIR (2009). Identifications were confirmed by Dr. Claudio Canepari, an authority on the family Coccinellidae from Italy.

## Results

### Tribe Scymnini Mulsant

Scymnini Mulsant 1846

Small; elongately oval, moderately convex; dorsal surface pubescent; coloration variable; head with clypeus straight anteriorly, with minute emarginations; antennae short, 8–11 segmented, terminal segments forming distinct club; eyes small, coarsely faceted, pubescent; maxillary palp with terminal segment cylindrical narrow or weakly expanded toward distal end; anterior pronotal margin weakly excavated; anterior margin of prosternum flat, straight never concealing mouth parts, prosternal process with or without longitudinal carinae; elytral epipleura very narrow, without any distinct foveae; tibial spurs present; tarsi trimerous or cryptotetramerous; abdomen with six visible sternite in male and female.

**Genitalia:** Male genitalia with tegmen short, narrow to broader, sipho elongated, narrow with hammer shaped siphonal capsule; female genitalia with genital plate long, narrow, or short, nearly round; spermatheca present, sign of interrogative shaped.

### Key to the Genera of the Tribe Scymnini

1. Antennae 10 or 11 segmented; prosternum with carinae; tarsus four-segmented; poscoxal line incomplete or complete; infundibulum present ***Scymnus* Kugelann.**
– Antennae pseudo-11-segmented; prosternum without carinae; tarsus three-segmented; postcoxal line incomplete; infundibulum absent ***Nephus* Mulsant.**

**Genus *Scymnus* Kugelann**

*Scymnus* (Kugelann 1794: 545; Mulsant 1846: 219, 1850: 965; Crotch 1874: 239; Korschefsky 1931: 115).

**Type species:** *Scymnus nigrinus* (Kugelann 1794: 548), by subsequent designation.

**Coloration:** Dorsal surface moderately pubescent; elytra coloration variable.

**Size and general shape:** Adult length 1–3 mm; width 1–2 mm; body oval, moderately convex.

**Head:** Clypeus with anterior margin truncate or slightly convex, emargination without setae; antennae 10 or 11-segmented, basal segment stout, about twice as long as wide, slightly curved, second segment slightly shorter than broad but equal in width to basal; third segment slender, about twice as long as broad, fourth and sixth about equal, either shorter than fifth but all about the same width, seventh and eighth segments slightly longer and broader, ninth segment much wider at apex than at base, 10th segment a little broader than long, 11th segment as long as 10th, rounded at apex, the last three or four segments forming a compact club; maxillary palpus with apical segment cylindrical, apex obliquely truncate.

**Thorax:** Pronotum with posterior margin medially deeply emarginated; prosternum straight anteriorly, carinate; elytral epipleura weakly narrow distally; tibial spurs absent; tarsal claws with an acute basal tooth.

**Abdomen:** Abdomen with six visible sternites; postcoxal line incomplete (subgenus *Scymnus*) str. or complete (subgenus *Pullus*).

**Male genitalia:** Median lobe symmetrical or asymmetrical, mostly shorter than paramere; siphon with siphonal capsule not complete hammer shaped, with adjacent arm shorter than opposite arm; trabes curved, mostly broader apically.

**Female genitalia:** Spermatheca hook shaped, elongated, basally rounded with very small accessory gland with short narrow duct.

**Type species:** *S.* *nigrinus* (Kugelann 1794).

### *Key to the Subgenera of the Genus* Scymnus

1. Antennae 10 segmented; postcoxal line incomplete, curved forward apically; male 5th and 6th abdominal sterna truncate or emarginate apically ***Scymnus* (*Scymnus*).**
– Antennae 11-segmented; postcoxal line complete, recurved apically, reaching base of first abdominal sternum; 5th and 6th abdominal sterna moderately to strongly emarginate and impressed posteriorly ***Scymnus* (*Pullus*).**

**Subgenus *Scymnus* (*Scymnus*) Kugelann**

**Head:** Antenna 10 or 11 segmented.

**Thorax:** Prosternum with two strong carinae.

**Abdomen:** Postcoxal line incomplete, curved forward apically; male 5th and 6th abdominal sterna truncate or emarginate apically.

**Subgenus *Scymnus* (*Pullus*) Mulsant**

**Head:** Antennae 11 segmented.

**Thorax:** Prosternum with distinct carinae.

**Abdomen:** Postcoxal line complete, recurved apically, reaching base of first abdominal sternum; 5th and 6th abdominal sterna moderately to strongly emarginate and impressed posteriorly.

***Scymnus* (*Scymnus*) *nubilus* Mulsant**

**Fig. 1.**
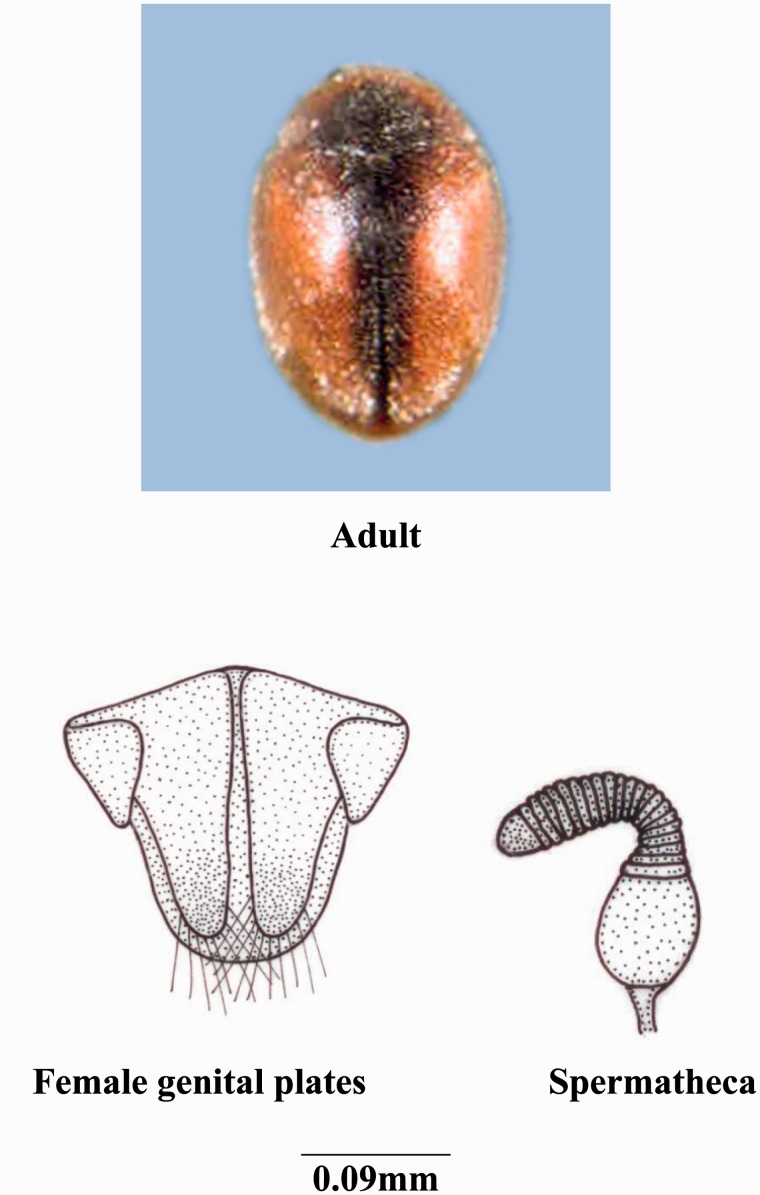
*Scymnus* (*Scymnus*) nubilus Mulsant.

**(****Fig. 1****)**

*Scymnus nubilus* (Mulsant 1850: 972, Bielawski1972: 293, Booth and Pope 1989: 359)*.*
*Scymnus* (*Scymnus*) *nubilus*: Korschefsky (1931: 143) (cat.).
*Scymnus curtisii* (Mulsant1850: 973), synonymized by Booth and Pope (1989: 351).
*Scymnus suturalis* (Motschulsky 1858: 120, Crotch 1874: 253, Korschefsky 1931: 144).
*Scymnus stabilis* (Motschulsky 1866: 426, Crotch 1874: 257, Weise 1900: 439, Korschefsky 1931: 144) (cat.).
*Scymnus lateralis* (Sicard 1913: 502, Korschefsky 1931:143 (cat.), Pang and Gordon 1986: 133); synonymized by Booth and Pope (1989: 360).

**Coloration:** Body dark brown, lighter in male, eyes brown, pronotum dark pitchy brown to black in middle, anterior, and lateral margins reddish brown and lighter, scutellum dark brown, elytra yellowish to reddish brown, with a dark brown to black sutural stripe starting from basal margin and gradually narrowed toward apex, lateral borders narrowly dark brown to black at middle, elytra and pronotum densely covered with short transparent yellow hairs ventral side reddish to yellowish brown, except pro-, meso- and metasterna and middle of abdominal segments dark brown and covered with fine hair and finely pitted.

**Size and general shape:** Adult length 1.6–2 mm; width 1–1.3 mm; body small and elongated-oval.

**Head:** Eyes large; labrum with anterior margin convex; ligula with straight margin; terminal segment of labial palp long pointed and converged.

**Thorax:** Anterolateral margin of pronotum without hairs; prosternal process with carinae posteriorly divergent; scuto-scutellar suture straight; tibia with dorsal margin distally with densely long hairs.

**Abdomen:** Second to fourth segments narrow medially except the terminal segment; postcoxal line incomplete running parallel to posterior margin to nearly 3/4th of its length and then gently recurved; terminal sternite with posterior margin with hairs entire.

**Male genitalia:** Sipho bifurcated and broader before the terminal end, terminal end broader and tetragonal, siphonal capsule with inner arm dorsally emarginated forming trigonal shape, outer arm straight and elongated expanded distally; tegmen with trigonal basal piece with no emargination, medial lobe short and broader; parameres straight elongated; trabes medially bended or curved.

**Female genitalia:** Genital plates cylindrical-elongated; lateral plates completely trigonal in shape; 10th tergite with straight margin; nodulus very short, cornu thick elongated slightly expanded distally; ramus totally absent; infundibulum present.

**Material examined:** Fifteen males, 16 females, Pakistan; Sindh: Tandojam, Sukkar, Mirpur Khas, Hyderabad, Karachi, Punjab: Jhang, Lyalpur, 13 April 2009; 22 March 2008 on brinjal, okra, cotton, leg., Khan, M. I. and Ali, M., lodged at Natural History Museum, Department of Zoology, University of Karachi. All male were lost due to attack of germs.

**Comparative note:** It resembles in external appearance with *hoffmanni* but differs this species from *nubilus* due to larger in size and the more thickened sutural stripe. The species *nubilus* belongs to the subgenus *Scymnus* (*Scymnus*), whereas the later one belongs to the subgenus *Scymnus* (*Neopullus*).

**Distribution:** India; Bangladesh; Sri Lanka; Nepal; Myanmar; China; Japan; Micronesia; Portugal; La Reunion; Africa Region; Pakistan: Sindh: Karachi, Hyderabad, Sukkar, Tandojam, Larkana, Mirpur Khas; Punjab: Lahore, Jhang, Faisalabad; Baluchistan: Quetta; Khyber Pakhtunkhwa: Peshawar.

### *Key to the Species of the SUBGENUS* Scymnus *(*Pullus*)*

1. Body large; with spots or patches; siphon with siphonal capsule slightly to deeply screw driver shaped **2.**
– Body small; without patches or spots; siphon hammer shaped
***Scymnus* (*Pullus*) *castaneus* Sicard.**
2. Sipho posteriorly pointed; basal lobe thick, compressed, posteriorly pointed; genital plate long triangular; lateral plate large **3.**
– Sipho posteriorly bifurcated; basal lobe expanded posteriorly with a deep cutting laterally on each side; genital plate short, triangular; lateral plate small
***Scymnus* (*Pullus*) *quadrillum* Motschulsky.**
3. Body brownish-black; each elytron with one black spot attached with one patch on each lateral side; sipho terminally with a pointed hook; trabes terminally fin-shaped ***Scymnus* (*Pullus*) *syriacus*.**
– Body brownish-yellow; each elytron with a glass shaped patch in basal half, two small spots on posterior half; siphon terminally straight; trabes terminally spoon shaped
***Scymnus* (*Pullus*) *coccivora* Ayyar.**

***Scymnus* (*Pullus*) *qaudrillum* Motschulsky**

**Fig. 2.**
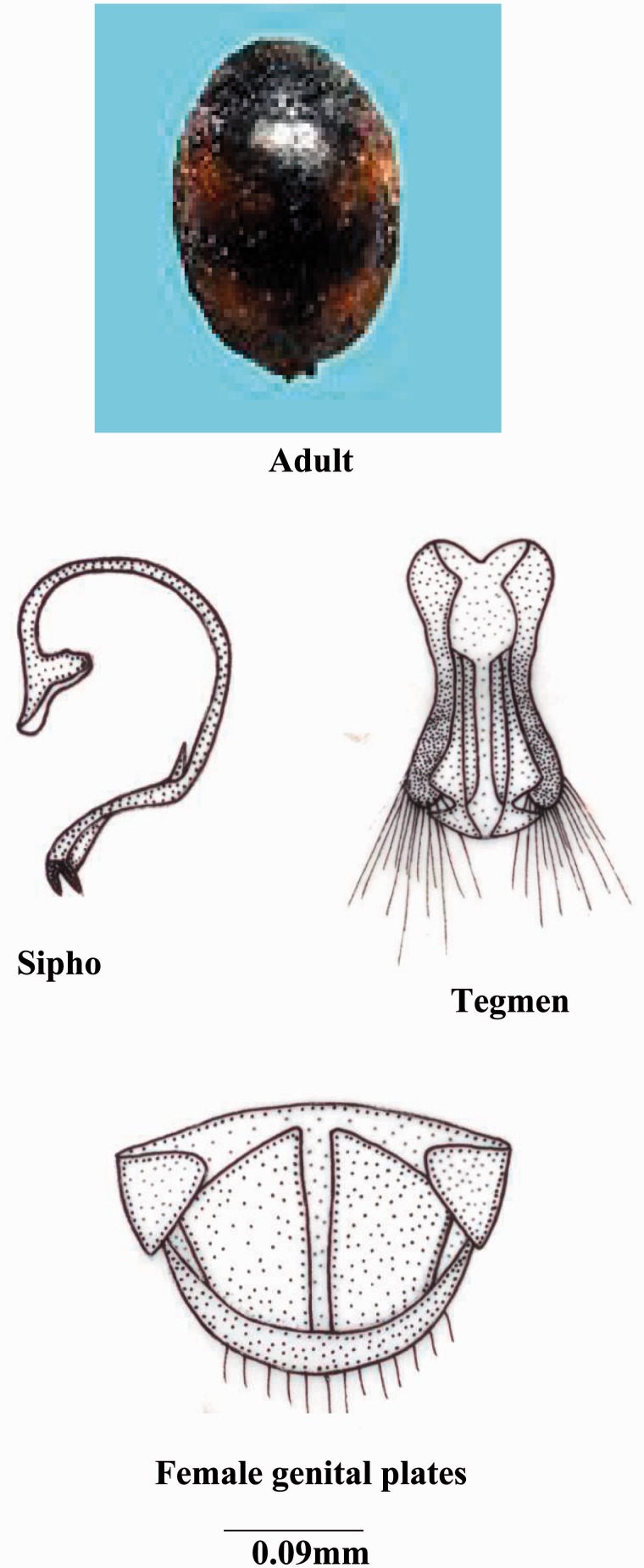
*Scymnus* (*Pullus*) qaudrillum Motschulsky.

**(****Fig. 2****)**

*Scymnus quadrillum* (Motschulsky 1858: 120, Crotch 1874: 252, Weise 1900: 436, Korschefsky 1931: 144).

**Coloration:** Body glabrous or pubescent; head brownish black; pronotum and elytra shiny dark to bluish in color, scutellum black, two reddish spots on each elytron; color and size variation in spots are common; ventral side brownish black.

**Size and general shape:** Adult length 1.6–2.0 mm; width 1.3–1.5 mm.

**Head:** Eyes large covering ¾th part of head; anterior margin of labrum convex; ligula expanded anteriorly with straight margin; labial palp with terminal segment small and pointed.

**Thorax:** Anterolateral margin of pronotum without hairs; prosternal process with carinae reaching to the anterior margin, anteriorly broad; scuto-scutellar suture narrow medially while broader proximally and distally; tibia with dorsal margin distally with small hairs.

**Abdomen:** Second to fourth segments narrow medially and broader laterally; fifth segment larger in both sexes; postcoxal process bifurcated; postcoxal line complete strongly v-shape; terminal sternite with posterior margin with few hairs.

**Male genitalia:** Sipho broader, thick, sclerotized and bifurcated terminally and bearing a hook at the starting of this thick part; siphonal capsule completely funnel shaped; basal piece elongated and triangular; parameres slightly diverted; median lobe knife shaped with a deep cutting laterally before the terminal end; trabes straight expanded apically.

**Female genitalia:** Genital plates larger and slightly tetragonal; lateral plates small and completely trigonal shaped; 10th tergite broader medially and narrow laterally.

**Material examined:** Eighteen males,14 females, Pakistan; Sindh: Tandojam, Sukkar, Mirpur Khas, Hyderabad, Karachi, Punjab: Jhang, Lyalpur,13 April 2009; 23 March 2008 okra, cotton, leg., Khan, M. I. and Ali, M., lodged at Natural History Museum, Department of Zoology, University of Karachi.

**Comparative note:** This species is similar in the external appearance with *taiwanus,* but in *taiwanus,* the four spots on elytra are arranged along the lateral margin, differs in shape, and somewhat the body coloration is also slightly variable. The species *qaudrillum* sometime exactly resembles *frontalis* having very little differences.

**Distribution:** India; Bangladesh; Sri Lanka; Thailand; Taiwan; India; Nepal; Vietnam; Laos; China; Pakistan: Sindh: Karachi, Hyderabad, Sukkar, Larkana, Mirpur Khas; Punjab: Lahore, Faisalabad; Baluchistan: Quetta; Khyber Pakhtunkhwa: Peshawar.

**Remarks:** It is newly recorded from Pakistan.

***Scymnus* (*Pullus*) *coccivora* Ayyar**

**Fig. 3.**
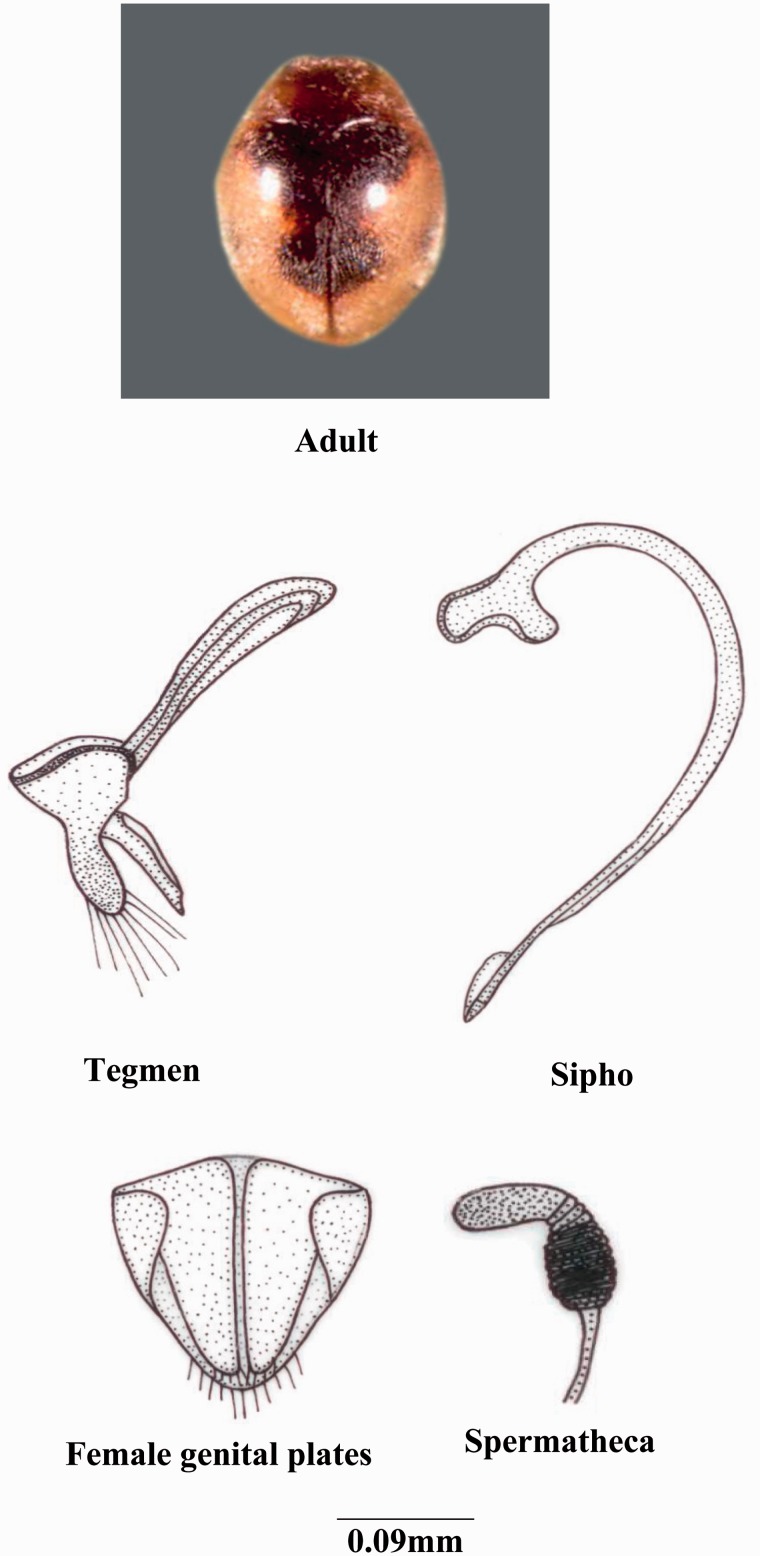
*Scymnus* (*Pullus*) coccivora Ayyar.

*Scymnus coccivora* (Ayyar 1925: 491).
*Scymnus* (*Pullus*) *coccivora* (Korschefsky 1931: 142).
*Pullus coccidivora* (Chelliah 1965: 165).
*Scymnus* (*Pullus*) *elegans* (Sicard 1929: 182, Korschefsky 1931: 142) (cat.).
*Scymnus* (*Pullus*) *elegans* var. clathratus (Sicard 1929: 182).
**(****Fig. 3****)**

**Coloration:** Body brownish yellow, pale golden yellow to yellowish brown, with dark purplish brown markings on elytra, elytral with a glass shape longitudinal spot on basal half, two small circular spots on posterior half, sometimes with only posterior spots present or uniformly yellowish brown, completely devoid of markings.

**Size and general shape:** Adult length 1.70–1.90 mm; width 1.20–1.30 mm; small, elongated-oval, moderately convex.

**Head:** Eyes small; anterior margin of labrum slightly notched; ligula with straight margin; terminal segment of labial palp small pointed and converged.

**Thorax:** Anterolateral margin of pronotum with few hairs; prosternal process with carinae broader toward posterior; scuto-scutellar suture fattened distally and narrower proximally; tibia with dorsal margin distally with minute hairs.

**Abdomen:** Second to fourth segments equal in size except the terminal segment; postcoxal line complete, semicircular; postcoxal process slightly notched; terminal sternite with posterior margin with hairs entire.

**Male genitalia:** Sipho terminally pointed with an additional membrane, anteriorly thick, siphonal capsule completely screw driver shaped; basal piece short, trigonal with thick margins; median lobe longer than parameres, terminally slightly upwarded; parameres short, spotted, straight; trabes with thick lateral margins, narrower proximally and broader medio-distally.

**Female genitalia:** Genital plates and lateral plates elongated and trigonal in shape; 10th tergite narrow medially with elongated lateral arms; spermathecal capsule with only elongated oval cornu, basally broader and oval, the part between cornu and basal region narrow and constricted.

**Material examined:** Fifteen males, 16 females, Pakistan; Sindh: Tandojam, Sukkar, Mirpur Khas, Hyderabad, Karachi, Punjab: Jhang, Lyalpur, 13 April 2009; 22 March 2008 on brinjal, okra, cotton, leg., Khan, M. I. and Ali, M., lodged at Natural History Museum, Department of Zoology, University of Karachi.

**Comparative note:** This species is similar sometimes in coloration and shape with *castaneus* but differs due to the presence of elytral glass shape longitudinal spot on basal half, two small circular spots on posterior half, sometimes with only posterior spots. In *castaneus,* spots are totally absent and also it is smaller in size than *coccivora*.

**Distribution:** India; Bangladesh; Sri Lanka; Malaysia; Pakistan: Sindh: Karachi, Hyderabad, Sukkar, Tandojam, Larkana, Mirpur Khas; Punjab: Lahore, Faisalabad (Lyalpur), Murre; Baluchistan: Quetta; Khyber Pakhtunkhwa: Peshawar, Swat; Kashmir.

***Scymnus* (*Pullus*) *castaneus* Sicard**

*Scymnus* (*Pullus*) *castaneus* (Sicard 1929: 180).

*Scymnus* (*Pullus*) *castaneus* (Korschefsky 1931: 142) (cat.).

**Fig. 4.**
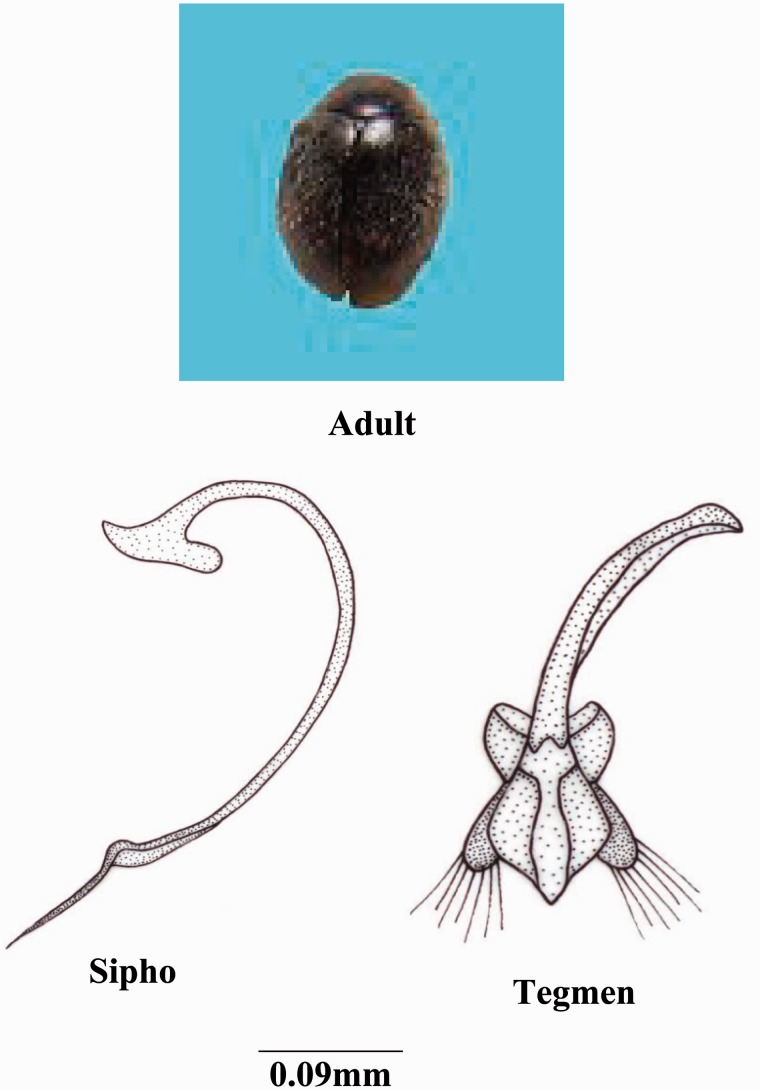
*Scymnus* (*Pullus*) castaneus Sicard.

**(****Fig. 4****)**

**Coloration:** Body dark reddish brown to dark brown, pronotum slightly darker; ventral side reddish brown to dark brown, legs yellowish brown; pronotum and elytra densely punctuate.

**Size and general shape:** Adult length 2.10–2.40 mm, width 1.60–1.90 mm; oval, moderately convex.

**Head:** Labrum with anterior margin slightly notched; ligula with straight margin; terminal segment of labial palp small pointed and converged.

**Thorax:** Anterolateral margin of pronotum bearing a group of long hairs; prosternal process with carinae strongly divergent posteriorly, anteriorly pointed; scuto-scutellar suture slightly straight, broader toward ends while narrower medially; tibia with dorsal margin distally with small hairs.

**Abdomen:** Second to fourth segments equal in size while last segment larger; postcoxal process slightly bifurcated; postcoxal line complete, broadly v-shaped; terminal sternite with posterior margin with hairs entire.

**Male genitalia:** Sipho elongated terminally with narrower and s-shaped end, siphonal capsule with opposite arm short, pointed and curved while adjacent arm straight, elongated and distally rounded; basal piece triangular; median lobe longer than parameres and broadly expanded medially; trabes thick proximally and narrow and curved distally.

**Material examined:** Fifteen males, Pakistan; Sindh: Tandojam, Sukkar, Mirpur Khas, Hyderabad, Karachi, Punjab: Jhang, Lyalpur, 13April 2009; 22 March 2008 on brinjal, okra, cotton, leg., Khan, M. I. and Ali, M., lodged at Natural History Museum, Department of Zoology, University of Karachi.

**Comparative note:** This species is similar sometimes in coloration and shape with *coccivora* but differs because it is larger in size, lways uniformly yellowish brown, completely devoid of markings but *coccivora* smaller in size and mostly markings on elytra are present.

**Distribution:** Bangladesh; India; Pakistan: Sindh: Karachi, Hyderabad, Tandojam, Sukkar, Larkana, Mirpur Khas; Punjab: Lahore, Jhang, Faisalabad (Lyalpur); Baluchistan: Quetta; Khyber Pakhtunkhwa: Peshawar.

**Remarks:** This species is also newly recorded from Pakistan.

***Scymnus* (*Pullus*) *syriacus* (Marsuel 1868)**

**Fig. 5.**
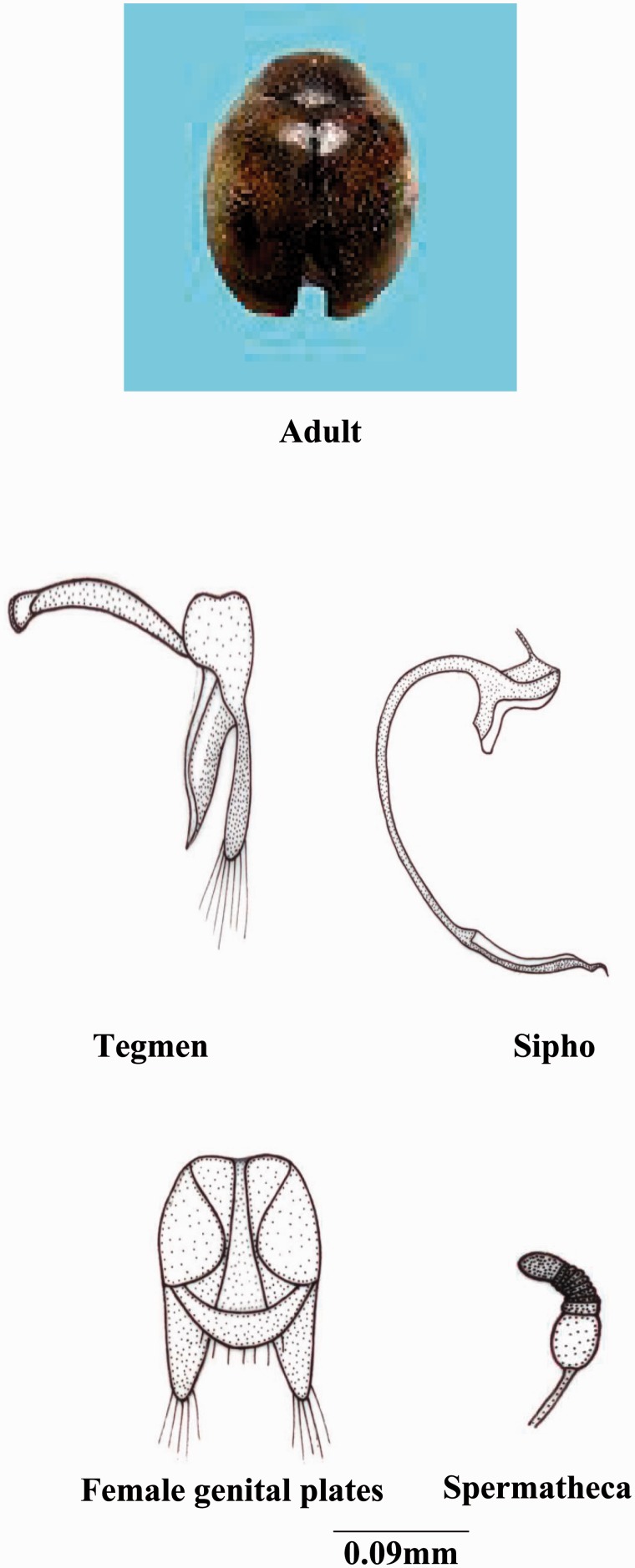
*Scymnus* (*Pullus*) syriacus (Marsuel).

**(****Fig. 5****)**

**Coloration:** Body reddish brown, pubescent, head, mouth parts, ventral side all black, each elytron with a large longitudinal black spot attached with one small brownish patch on each side.

**Size and general shape:** Adult length 2–2.3 mm; width 1–1.2 mm; rounded-oval, convex.

**Head:** Labrum with anterior margin slightly notched; ligula with straight margin; terminal segment of labial palp small pointed and converged.

**Thorax:** Anterolateral margin of pronotum without hairs; prosternal process with well-developed carinae, narrow anteriorly, reaching the anterior margin; scuto-scutellar suture curved posteriorly; tibia with dorsal margin distally bearing long hairs.

**Abdomen:** Second to fourth segments equal in size except the terminal segment; postcoxal line complete, strongly v-shaped; postcoxal process with anterior margin broader, straight; terminal sternite bearing long setae medially while short setae laterally in male.

**Male genitalia:** Sipho narrow, distally flat, pointed, siphonal capsule with opposite arm long, curved while adjacent arm short, pointed; basal piece triangular, broader than long; median lobe thick, slightly longer than paramere; paramere narrow proximally, expanded distally; trabes curved, expanded medially, terminal end bearing fin shaped process.

**Female genitalia:** Genital plate long, broader; lateral plate triangular, deeply curved inside; 10th tergite broader medially, narrow laterally; spermatheca with base rounded-oval, cornu long, narrow deeply curved.

**Distribution:** Iran; Afghanistan; Egypt; Pakistan.

**Remarks:** It is newly recorded from Sindh Province of Pakistan.

**Genus *Nephus* Mulsant**

*Scymnus* (*Nephus*) (Mulsant 1846: 237).

*Nephus* (Motschulsky 1866: 425).

Type species: *Coccinella quadrilunulatus* Illiger*,* 1798, by subsequent designation of Korschefsky (1931).

*Sidis* (Mulsant 1850: 975)

Type species: *Scymnus* (*Sidis*) *binaevatus* Mulsant, by subsequent designation of Korschefsky (1931).

*Nephus* (*Bipunctatus*) (Fürch 1987: 66).

Type species: *Scymnus bipunctatus* Kugelann, 1794.

*Nephus* (*Geminosipho*) (Fürch 1987: 68).

Type species: *Nephus bielawskii* Fürsch 1965.

*Nephus* (*Parascymnus*); Fürch, 1987: 66 (*Parascymnus* Chapin, 1965b downgraded).

Type species: *Parascymnus palauensis* Chapin, by original designation.

Aponephus Booth‡

Type species: *Aponephus lentiformis* by original designation. Syn. nov.

**Coloration:** Dorsal surface pubescent, brownish black, sometime with spots.

**Size and general shape:** Adult length 1.6–1.8 mm; width 1.2–1.4 mm oval, moderately convex.

**Head:** Clypeus with anterior margin slightly truncate, emarginations with minute setae; antenna pseudo-11 segmented or 10 or 11 segmented, first and second segments completely tightly jointed together, basal segment moderately stout, curved, second segment separated from first by a false suture, not always visible, half as long as broad but equal in width to basal, third segment a little longer than broad, fourth, fifth, and sixth segments of same width, the fifth slightly shorter than fourth or sixth, seventh, eighth, and ninth segments progressively slightly longer and wider, 10th segment slightly longer than broad but nearly quadrate, 11th segment half as long as 10th, almost hemispherical; maxillary palpus with apical segment cylindrical, apex obliquely truncate.

**Thorax:** Pronotum with posterior margin medially deeply emarginated; prosternum slightly produced anteriorly; prosternal process with carinae; elytral epipleura weakly narrow distally; tarsi three segmented; tibial spurs absent; claw with acute basal tooth.

**Abdomen:** Abdomen with six visible segments; postcoxal line incomplete.

**Male genitalia:** Median lobe symmetrical distinctly narrow, shorter than paramere; sipho with siphonal capsule hammer shaped but opposite arm distinctly broader; trabes slightly expanded apically.

**Female genitalia:** Spermatheca weakly hook shaped, deeply elongated with cornu and basal part distinctly separated with very small accessory gland with very short duct.

***N. regularis* (Sicard)**

*Scymnus* (*Nephus*) *regularis* (Sicard 1929: 183, Korschefsky, 1931: 144 (cat.))

*N. regularis*: (Chelliah 1965: 166, Pang and Gordon 1986: 133).

**Fig. 6.**
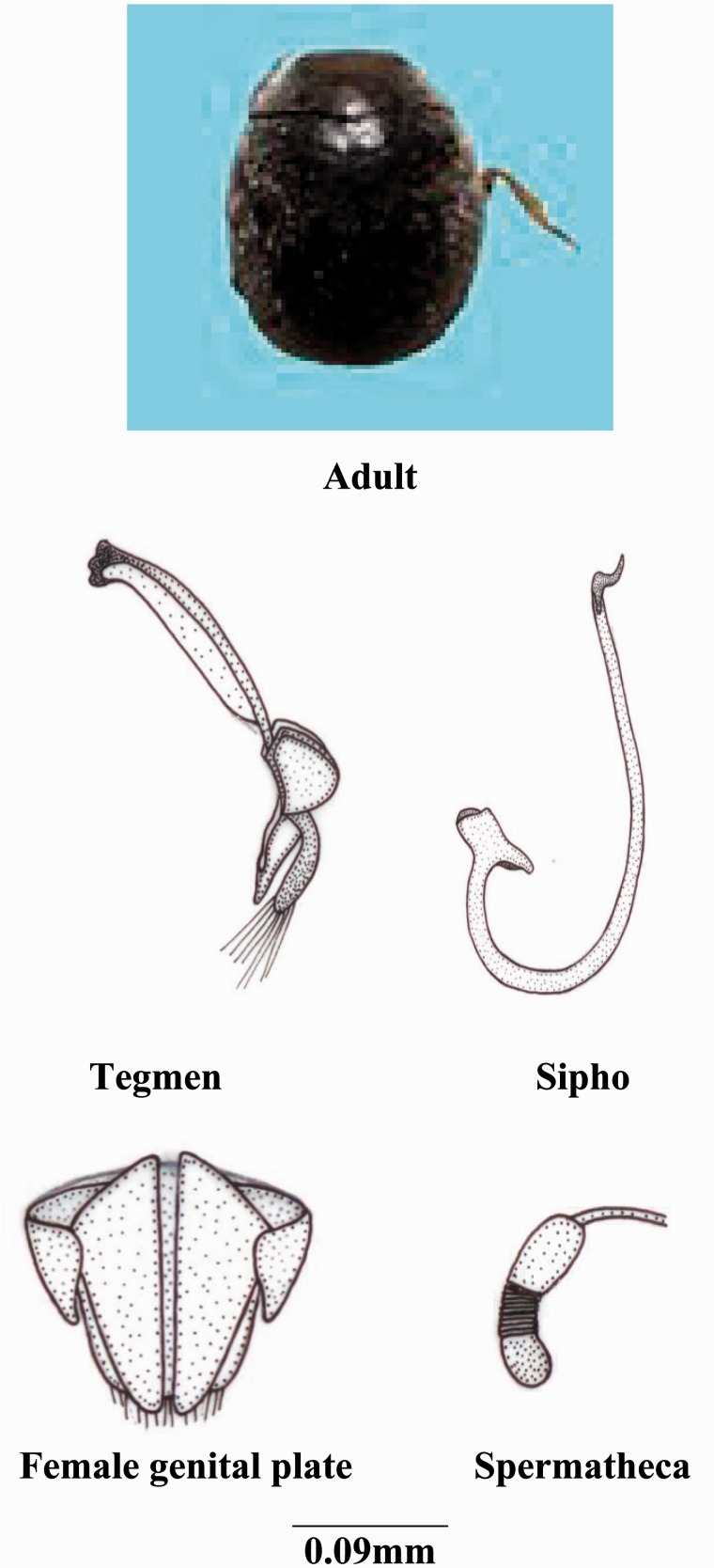
*N. regularis* (Sicard).

**(****Fig. 6****)**

*Scymnus* (*Nephus*) *regularis* (Sicard 1929: 183).

**Coloration:** Head and pronotum yellowish brown; elytra light yellowish brown, area along basal margins and suture darker, often with a distinct dark patch.

**Size and general shape:** Adult length 1.6–1.8 mm; width 1.2–1.4 mm; short oval, moderately convex.

**Head:** Labrum large, with convex anterior margin; ligula with anterior margin slightly notched; labial palpus with terminal segment deeply pointed.

**Thorax:** Anterolateral margin of pronotum with minute hairs; prosternal process broader than long, without carinae and finely punctuate; tibia elongated, narrow, slightly oval with minute hairs dorsally.

**Abdomen:** Second to third, terminal segments large, fourth to fifth smaller; postcoxal process deeply notched; postcoxal line incomplete, parallel to posterior margin of first abdominal ventrite for up to 4/5^th^ of its length and then very slightly recurved; last segment with small haors throughout.

**Male genitalia:** Sipho thick proximally and slightly narrow distally bearing terminally a hook just like the sign of interrogation and medially passing a line from mid to end of sipho, siphonal capsule with opposite arm short, broader and tetragonal shaped while adjacent arm narrow and spoon shaped distally; tegmen with basal piece slightly tetragonal with thick margins; medial lobe shorter than parameres with right lateral margin truncate terminally (cutting); trabes narrow proximally, medially expanded and terminally upwarded and saw like.

**Female genitalia:** Genital plates elongated and trigonal shape; lateral plates small and also trigonal; basal portion of spermatheca elongated and oval, cornu sclerotized.

**Material examined:** Fifteen males and 33 females, Pakistan; Sindh: Tandojam, Sukkar, Mirpur Khas, Hyderabad, Karachi, Punjab: Jhang, Lyalpur, 13April 2009; 22 March 2008 on brinjal, okra, cotton, leg., Khan, M. I. and Ali, M., lodged at Natural History Museum, Department of Zoology, University of Karachi.

**Comparative note:** This species resembles with *tagiapatus* in external appearance including the triangular brownish spot at elytral basal margins. The main difference is that in reguralis this spot is small only limited to the anterior basal part of elytra, but in *tagiapatus,* it is extended to the second and third parts along the medial junction as well as along lateral margins reaching to the middle of elytra.

**Distribution:** China; India; Pakistan: Sindh: Karachi, Hyderabad, Tandojam, Sukkar, Mirpur Khas; Punjab: Lahore, Faisalabad (Lyalpur); Baluchistan: Quetta; Khyber Pakhtunkhwa: Peshawar.

## Discussion

The study of male genitalia of the minute-sized species of the genus *Scymnus* was more difficult and for the first time proved to be final valid criteria for the identification at species level for any type of insect (Wilson 1927). This study helped to make the present investigation more valid by giving more focus on male genitalia of Coccinellids. Coccinellidae occurring in America north of Mexico, among which 57 genera (including six genera of the Scymnini) and 475 species (including above 90 species of Scymnini) were treated in a precise systematic way. Keys to all taxa, descriptions of the higher taxa, species diagnoses, synonymies, and host records were also included (Gordon 1985). The findings of this investigation supported this study in case of diagnostic characters, description of taxa and construction of identification keys to different groups of taxa of the family Coccinellidae. The species of the *Scymnus bipunctatus* group, overview of the genera and subgenera of the tribe Scymnini, and classification of the family Coccinellidae including six subfamilies with the addition of a new subfamily were given from West-Palaearctic region (Fürch 1987), which supports the classification of the Coccinellids including the tribe Scymnini of the present investigation. The annotated checklist of the Coccinellidae of the Indian Subregion covers the Coccinellids of India, Pakistan, Bhutan, Nepal, Sri Lanka, Bangladesh, and Myanmar and is considered to be more informative for the recent taxonomists of Coccinellids specially of these regions. In this checklist, a detailed geographical distributions and type depositories were mentioned. The author also recorded *N.* *reguralis* Sicard, *P.* *murreensis* Ahmad, *P*. *simmondsi* Ahmad, *Scymnus* (*Pullus*) *coccivora* Ayyar, *S*. (*Neopullus*) *fuscatus* Boheman, and *S*. (*Scymnus*) *nubilus* from Pakistan (Poorani 2002). This study supports all the findings, specially the identification and confirmation of Coccinellids studied in this study.

The works related to systematic, morphology, and distribution (Ahmad 1966, 1968; Inayatullah and Siddiqui 1978, 1979; Inayatullah 1980; Hashmi and Tashfeen 1992; Irshad 2001a,b, 2003; Rafi et al. 2005; Ali 2013) from Pakistan support the identification, preys with reference to host plants, and distribution of Coccinellids including the members of the tribe Scymnini carried out in this study, but very rare systematic works of the Coccinellids were found according to the viewed and cited literatures of this study. These findings confirm the presence of *Scymnus* (*Scymnus*) *nubilus* Mulsant, *Scymnus* (*Pullus*) *coccivora* Ayyar, *N. regularis* (Sicard) also in the Sindh Province of Pakistan, but this investigation is unique than the other findings, because it presents few new records such as *Scymnus* (*Pullus*) *syriacus* Marsuel, *Scymnus* (*Pullus*) *castaneus* Sicard, and *Scymnus* (*Pullus*) *quadrillum* Motschulsky.
